# Gene expression variation explains maize seed germination heterosis

**DOI:** 10.1186/s12870-022-03690-x

**Published:** 2022-06-20

**Authors:** Jiong Wan, Qiyue Wang, Jiawen Zhao, Xuehai Zhang, Zhanyong Guo, Desheng Hu, Shujun Meng, Yuan Lin, Xiaoqian Qiu, Liqin Mu, Dong Ding, Jihua Tang

**Affiliations:** 1 National Key Laboratory of Wheat and Maize Crop Science, College of Agronomy, Henan Agricultural University, Zhengzhou, 450002 China; 2 The Shennong Laboratory, Zhengzhou, 450002 China

**Keywords:** Maize, Seed germination, Heterosis, Gene regulation

## Abstract

**Background:**

Heterosis has been extensively utilized in plant breeding, however, the underlying molecular mechanism remains largely elusive. Maize (*Zea mays*), which exhibits strong heterosis, is an ideal material for studying heterosis.

**Results:**

In this study, there is faster imbibition and development in reciprocal crossing Zhengdan958 hybrids than in their parent lines during seed germination. To investigate the mechanism of heterosis of maize germination, comparative transcriptomic analyses were conducted. The gene expression patterns showed that 1324 (47.27%) and 1592 (66.44%) of the differential expression genes between hybrids and either parental line display parental dominance up or higher levels in the reciprocal cross of Zhengdan958, respectively. Notably, these genes were mainly enriched in metabolic pathways, including carbon metabolism, glycolysis/gluconeogenesis, protein processing in endoplasmic reticulum, etc.

**Conclusion:**

Our results provide evidence for the higher expression level genes in hybrid involved in metabolic pathways acting as main contributors to maize seed germinating heterosis. These findings provide new insights into the gene expression variation of maize embryos and improve the understanding of maize seed germination heterosis.

**Supplementary Information:**

The online version contains supplementary material available at 10.1186/s12870-022-03690-x.

## Background

Heterosis or hybrid vigor, refers to the phenomenon that hybrid progeny over one or both parents in many traits from quality traits to biomass, grain yield, growth rate, resistance to biotic and abiotic stress, growth potential, and adaptability [1, 2]. The utilization of heterosis has successfully improved yield and quality in many crops, however, its molecular mechanisms remain enigmatic [3, 4]. To explain the genetic bases of heterosis, three major theories, namely dominance, over-dominance, and epistasis, were established. The dominance model believes that the better performance of hybrids results from the aggregation of favourable alleles of both parents [5–7], among which the “good genes” pyramid in F_1_ were considered the key contributor to heterosis [8, 9]. The over-dominance hypothesis holds that the interaction between heterozygous alleles is greater than the homozygous alleles [10, 11]; while epistasis refers to the interaction between non-alleles on chromosomes [12, 13].

In the last decade, the gene expression regulation network has been made to dissect the genetic basis of heterosis with the rapid development of bioinformatics technology [14–16]. The additive and non-additive gene expression is a mixture of heterosis causal and effects [17]. The reason why these genes expression as the given model is determined by up-stream divergences, including cis-elements variations, trans-acting factor divergence, and divergent epigenetic effects [18–20]. Cis-element variation is one reason for heterosis since the gene expression of hybrids was under the same trans-action factor environment. It has been revealed that alleles from parental lines may be controlled by the specific binding cis-elements, resulting in allelic-specific expression [21]. In hybrids, the divergent trans-action factors from one parental line may influence the cis-elements, existing in the promoter, of the allele from another parental line, resulting in gene different expression [22]. The transcription factors may also function by forming complexes, it added another layer of trans regulation divergence that combination of two or more transcription factors, or the competing binding of co-regulators, will change the DNA binding specificity [23]. It has been reported that Epigenetics associated with distinct changes in the DNA methylation patterns may affect heterosis too [20, 24, 25].

Allele-specific expression (ASE, also called allelic expression or allelic imbalance), describes the expression variation between the two haplotypes in a diploid genome that showed heterozygous sites. Genome-wide ASE was caused by cis-elements variants, genome imprinting, and nonsense-mediated decay [26, 27]. The interactions between trans and cis-acting factors and their diversity further enhanced the complexity of ASE [28]. Allelic expression of multiple genes was considered to correlate to heterosis [29]. The extreme ASE, specific parental expression (SPE), was considered associated with the dominant effect [30–32].

Seed germination is a fundamental process, which directly influences the development of maize plants and further affects grain yields. During the early stage of germination, seeds absorb water rapidly from the field. Secondly, the metabolic process, generating a large number of energies, is reinitiated with various enzymes and hormones. And most of the energy, such as ATP, is provided by the glycolysis and the TCA cycle. Finally, the radicle breaks through the seed coat and then activates the establishment of seedlings [33]. It is suggested that F1 hybrid seeds show superior performance compared to their inbred parental lines in terms of seed germination [34]. It has also been proved that the embryo expansion rate at the early stages of maize seed germination is one of the heterosis traits [35].

Seed germination is a critical stage in plant life cycle that directly determines the establishment of the seedlings. Previous studies have proved that F_1_ hybrid seeds have a better performance over both parents during the germination stage, however, its molecular mechanism is still unclear [36]. Here, we performed a comparative transcriptome analysis of reciprocal cross for Zhengdan958and their parental lines. The main objectives of this study were to: 1) a genome-wide analysis of ASE for seed germination was performed; 2) and some key genes were validated by RT-qPCR. The results will provide new insight into gene expression variation for seed germination heterosis.

## Results

### Maize seed germination is a trait with heterosis

**Fig. 1:**
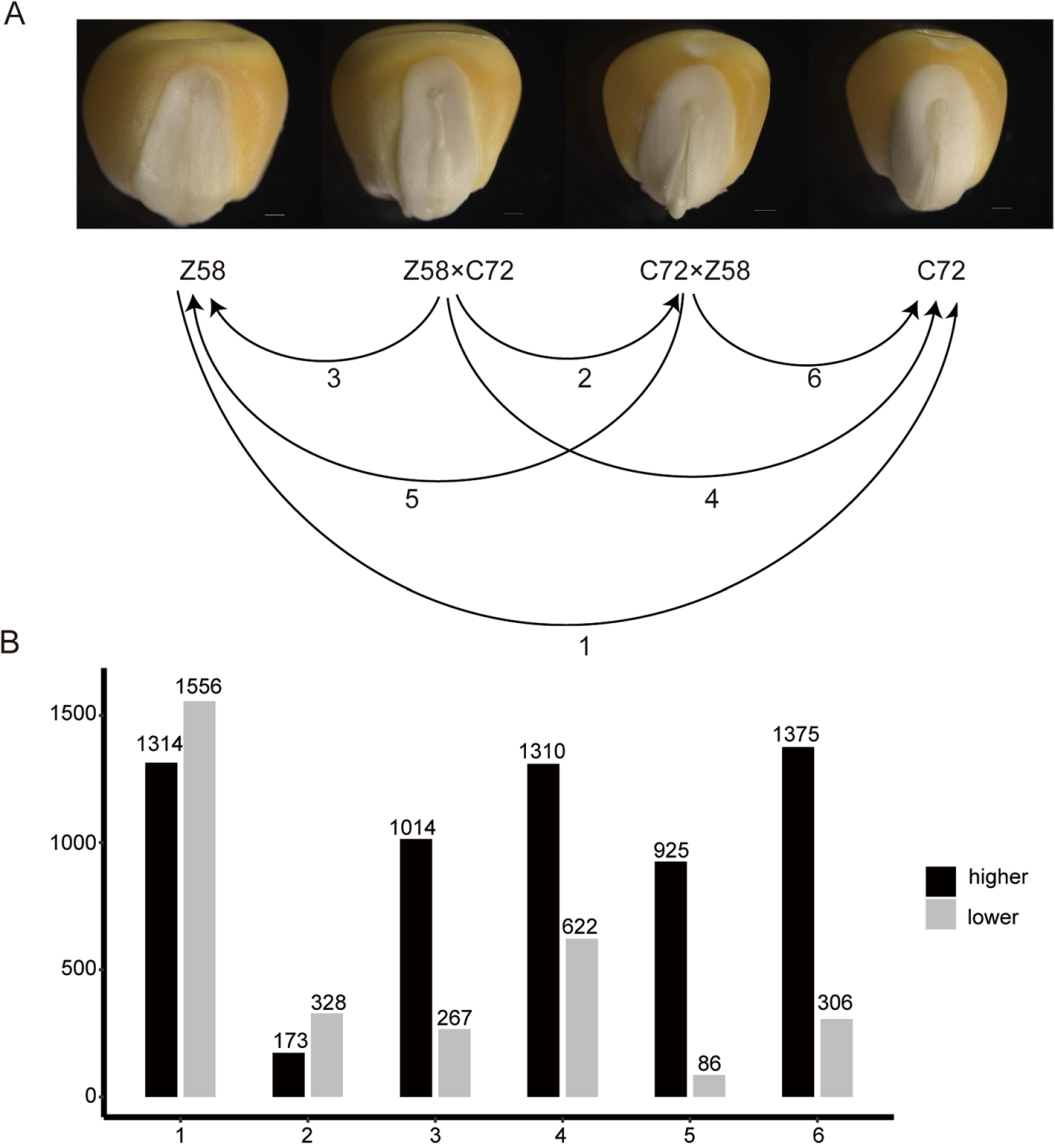
Phenotypic observations of maize embryos imbibition and overview of differential gene expression. **A** The transcriptomes of maize embryos of 24 hours imbibition of Zhengdan958 and their parental lines (C72 and Z58) were compared. Black arrows point to the control. **B** The differently expressed genes of six pairwise comparisons of the four genotypes are shown. These numbers of X axis correspond to those depicted in (A)

Maize seed germination or seedling establishment is considered to be a fundamental and critical process for plant growth and development [34, 35, 37]. The first stage of seed germination is embryos imbibition, at this stage, embryos were germinated under the support of nutrition stored in endosperm. It has bare communication with outside environments before the radicle grows out of the tip. It was shown that at this stage, the reciprocal crossing hybrids were fully imbibition, with radicle protrusion initiated earlier than in the two parental lines (Fig.1A). The divergence was more pronounced after 48 hours imbibition (Fig.S1A). We also performed a time-course of germination with 120 seeds for each genotype. It showed that approximately 90% of hybrid seeds showed visible radicle emergence after 36 hours germination compared with only 7 and 32% for Z58 and C72, respectively (Fig.S1B).

### Introduction of RNA seq data

A total of 12 germinating embryos samples were collected for RNA-seq profiling, including four genotypes (Reciprocal-crosses and two parental lines), each with three biological replicates. Approximately 51–86 million clean reads were obtained for each sample (Table 1), after low-quality reads were filtered, more than 98% clean reads mapped to the maize B73 reference genome (B73_RefGen_v4).

**Table 1:** Overview of RNA-seq data

Sample Name	Raw Reads	Clean Reads	Clean Reads Rate (%)	CleanQ30	Mapped Reads	Mapped Rate (%)
Z58-1	57,761,405	51,101,515	88.47	93.90;93.49	5,026,345,015	98.36
Z58-2	93,888,829	86,546,723	92.18	90.63;88.68	8,538,699,691	98.66
Z58-3	69,831,586	63,400,097	90.79	91.63;91.40	6,270,903,594	98.91
C72-1	63,556,262	58,160,335	91.51	88.45;89.53	5,722,395,361	98.39
C72-2	68,348,998	61,842,173	90.48	90.43;88.75	6,084,032,980	98.38
C72-3	67,664,143	63,387,769	93.68	90.43;91.73	6,246,230,757	98.54
Z58 × C72-1	75,728,777	70,049,119	92.5	92.83;90.29	6,915,949,519	98.73
Z58 × C72-2	58,642,488	53,024,538	90.42	90.33;89.92	5,238,294,109	98.79
Z58 × C72-3	67,336,587	61,114,686	90.76	89.43;85.13	6,016,740,837	98.45
C72 × Z58-1	83,526,998	77,354,353	92.61	91.06;91.53	7,607,800,618	98.35
C72 × Z58-2	77,185,580	71,458,410	92.58	92.98;90.25	7,024,361,703	98.3
C72 × Z58-3	67,380,639	63,182,825	93.77	92.61;92.03	6,220,980,950	98.46

RNA-seq reads information for the maize embryos samples

### Differentially expressed gene identification

To achieve a comprehensive overview of differential gene expression (DEGs), it was identified between all possible (*N* = 6) pairwise comparisons of the four genotypes. When controlling FDR at 5%, 2870 genes were significantly differentially expressed between parental lines after setting a threshold (log2 fold change> ±1). In the comparison between the reciprocal hybrids, 501 specific genes were differentially expressed. The overall number of genes differentially expressed between the four hybrid-inbred line comparisons were estimated to be ranged from 1011 to 1932 genes (Fig.1B). Thus, the level of expression divergence between inbred-hybrid comparisons is higher than the comparison of the reciprocal hybrids and lower than the comparison of the two parental inbred lines. Interestingly, among the genes which differentially expressed between parental lines and reciprocal hybrids, the expression of the majority genes (67.81 to 91.49%) in reciprocal hybrids is higher than that in the parental lines. It has been suggested that differential gene expression among hybrids and their parents plays important role in heterosis [8, 38]. Hence, these higher expressed genes may contribute more to heterosis during maize seed germination.

### Inheritance classifications

**Fig. 2:**
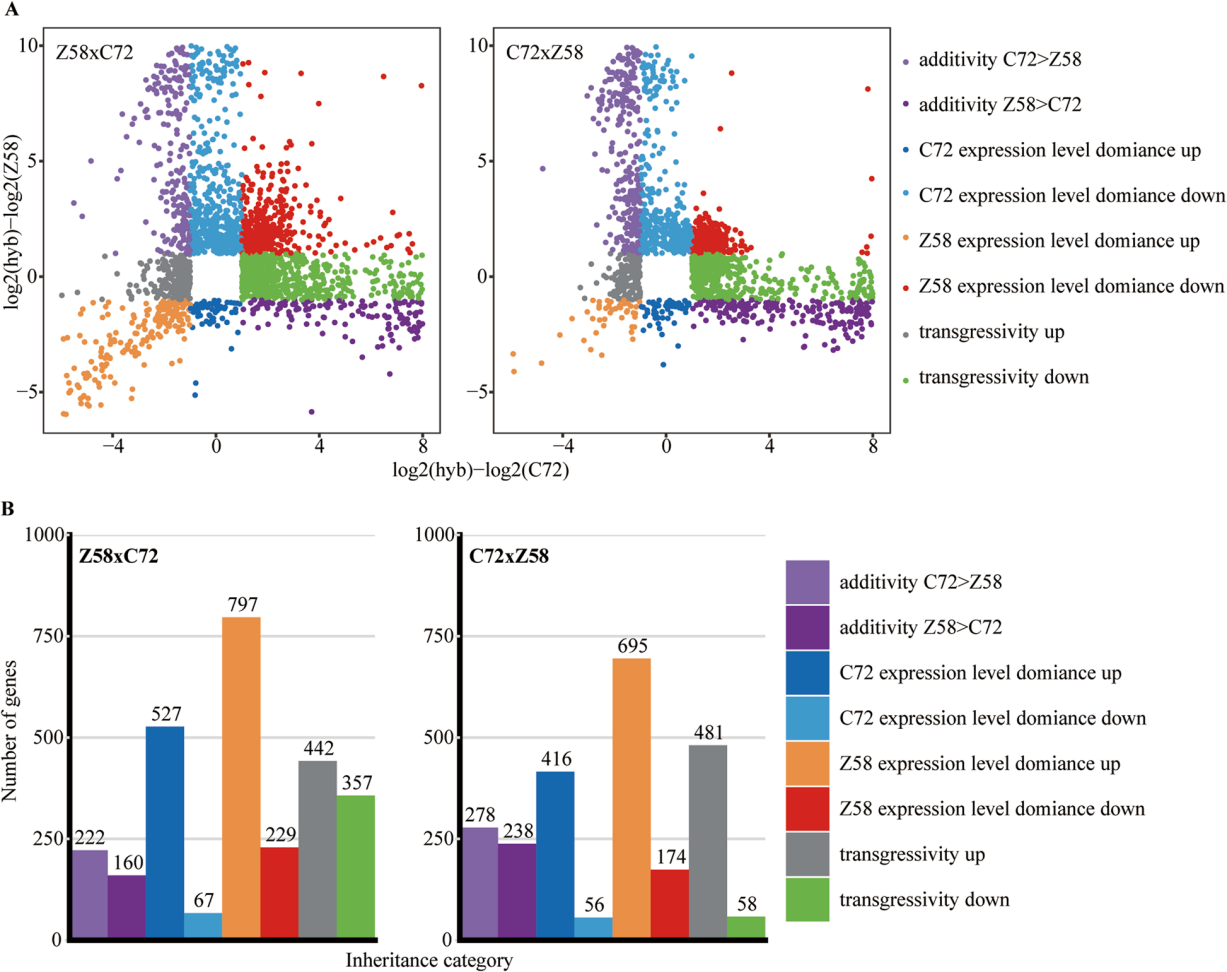
Inheritance classification of gene expression in Zhengdan958 and Zhengdan958 reciprocal-cross. **A** The scatterplots show the gene expression classification between reciprocal hybrids and their parental lines (C72 on the x axis and Z58 on the y axis). **B** The bar graphs show the number of genes in each expression inheritance category for Z58 × C72 and C72 × Z58 hybrids

According to comparing the expression level of hybrids and their parental lines, the genes divergently expressed between hybrids and parental lines were categorized into eight classes (Fig.2A). The detailed list was shown in Table S1. There are 2801 DEGs between Z58 × C72 and either parental line. 222 (7.93%) were classified as additivity C72 > Z58, 160 (5.71%) were additivity Z58 > C72, 527 (18.8%) were C72 expression level dominance up, 67 (2.39%) were C72 expression level dominance down, 797 (28.5%) were Z58 expression level dominance up, 229 (8.18%) were Z58 expression level dominance down, 442 (15.8%) were transgressivity up, 357 (12.7%) were transgressivity down. Based on this classification scheme, 1324 genes (47.27%) of divergently expressed genes between hybrid and parental lines were abundant in C72 or Z58 expression level dominance up category. 442 (15.78%) genes exhibited transgressivity up levels. A similar result was obtained in C72 × Z58 (Fig.2). Furthermore, both in Z58 × C72 and in C72 × Z58, the most abundant type included Z58 expression level dominance up (approximately 50% of divergent expression genes) (Fig.2B). This suggests that the majority (63%) of the DEGs are expressed at parental dominance up or higher levels in the hybrid. These results indicated that the genes showing parental dominance up or higher levels pattern might correlate with the observed seed germinating heterosis.

### Identification of allele-specific expression genes

**Fig. 3:**
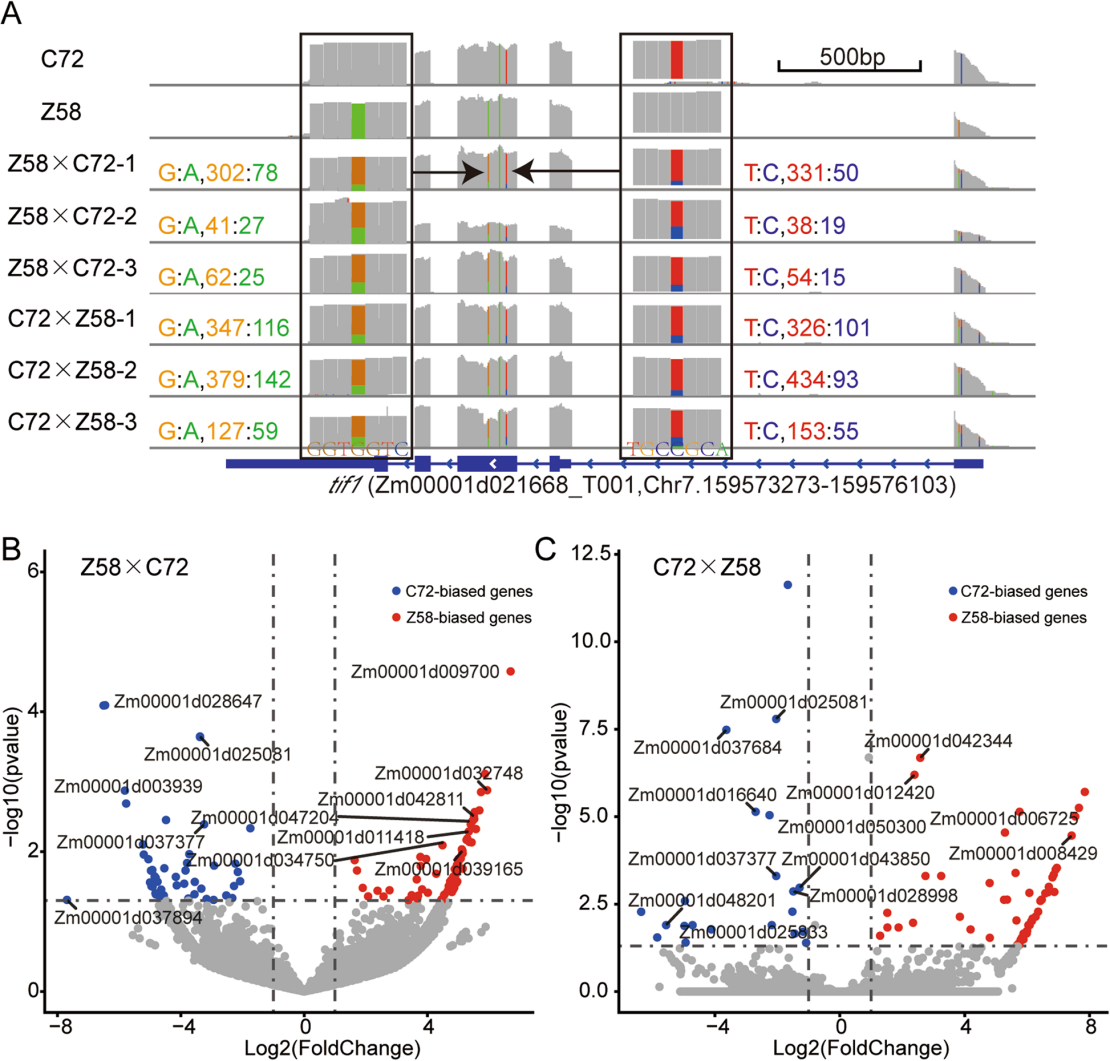
The characterization of Z58-biased and C72-biased ASE genes. **A** The IGV highlighting the read coverage on SNP of tif1 of C72 biased genes is shown. **B**,** C** The ASE genes in Z58 × C72 (B) and C72 × Z58 (C) are showed in volcano plots

It has been suggested that ASE, or imbalance between the expression levels of two parental alleles in the hybrid, was associated with heterosis [39]. To further explore the potential relationship between ASE and heterosis, genome-wide analysis of ASE was performed by comparing the reads ratio of the parental alleles in the transcriptome scale. The gene *Zm00001d021688* encodes translation initiation factor, which showed a G-to-A and a T-to-C base substitution at position chr7-159,574,201 and chr7-159,574,267, respectively (Fig.3A). The genes and the allelic ratio with statistic test are listed in Table S2. A total of 116 and 579 genes showed ASE in Z58 × C72 or C72 × Z58, respectively (Fig.3B, C). Out of the 116 ASE genes in Z58 × C72, 41 of which overlap with the DEGs. Out of the 579 ASE genes in C72 × Z58, 108 of which overlap with the DEGs. Cis-acting regulation refers to a conserved unbalanced allelic expression between parents and hybrids. Interestingly, for the 41 ASE genes in Z58 × C72 and 108 ASE genes in C72 × Z58, the allelic ratio in hybrid is similar to the parental proportions. This means that the ASE genes intersecting with DEGs are mainly due to cis-acting regulation.

### Analysis of functional enrichment of gene ontology

**Fig. 4:**
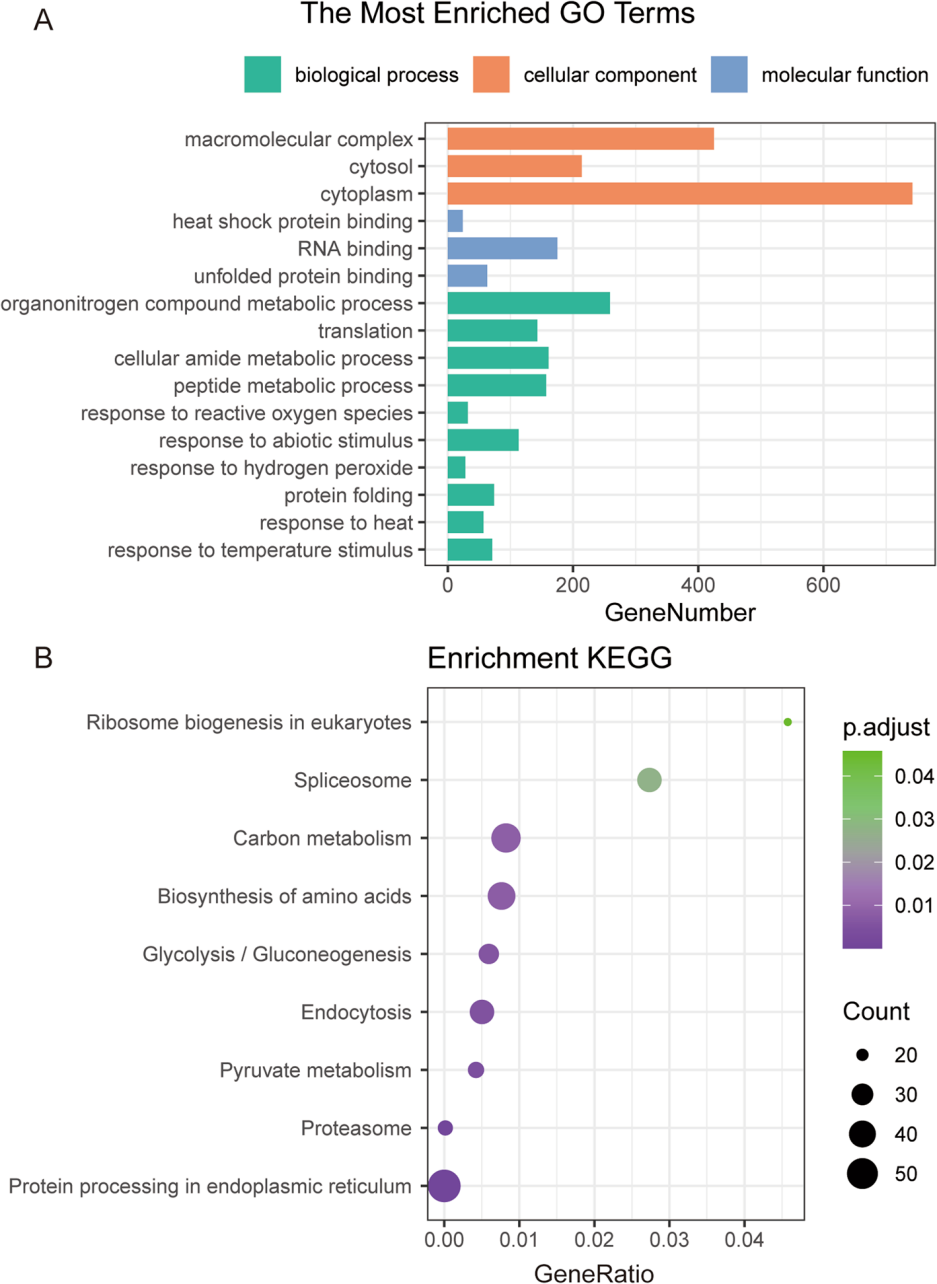
GO and KEGG enrichment analysis of parental dominance up or higher levels genes. **A** The top 20 GO term analysis of the shared genes. **B** The significant KEGG pathways of the shared genes

Almost half of the DEGs were in the parental dominance up level category. The distribution of genes in categories was similar to what has previously been observed for hybrids [18]. Consequently, these genes classified as parental dominance up and transgressivity up level may play important roles in heterosis. These genes were enriched to different classes and further analyzed using the AgriGOv2 database. The top 20 significantly enriched GO terms were shown in Table S3. In the biological process category, the most represented GO terms were response to temperature stimulus, response to heat, and protein folding. Most of the molecular function-related genes were involved in unfolded protein binding, RNA binding, and heat shock protein binding. For the cellular constituent category, the most terms were cytoplasm, cytosol, and macromolecular complex (Fig.4A).

The KEGG analysis indicated that the identified genes were mainly enriched in protein processing in endoplasmic reticulum, proteasome, pyruvate metabolism, endocytosis, glycolysis/gluconeogenesis, and carbon metabolism (Fig.4B). Notably, these pathways are significantly associated with metabolic regulation (Table S4).

### RT-qPCR confirmation

**Fig. 5:**
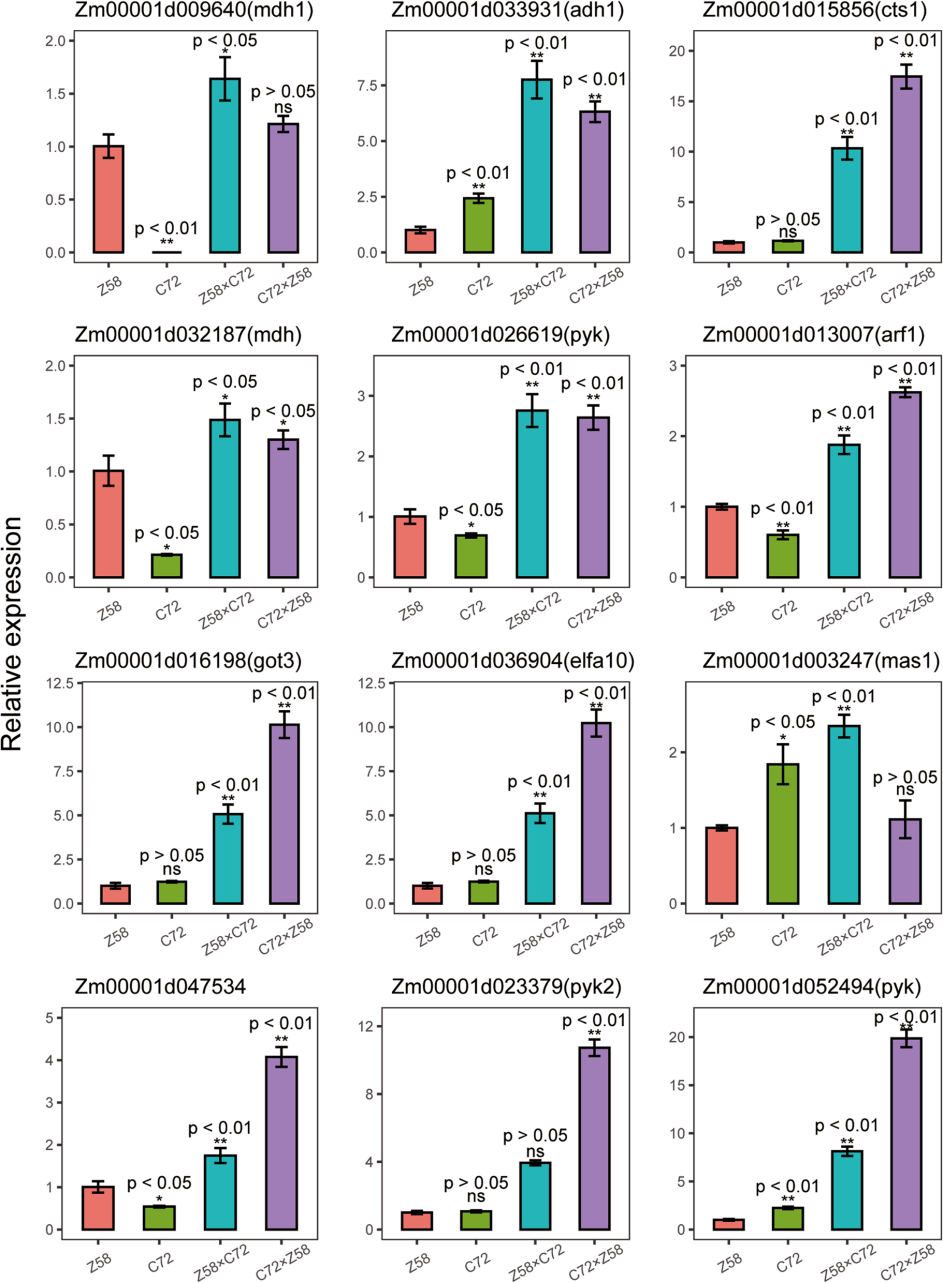
The relative expression levels of twelve genes involved in metabolic pathways as assessed by RT-qPCR

A subset of DEGs involved in metabolic pathways during maize seed germination was selected for RT-qPCR validation. These genes were mainly binned parental expression level dominance up category or above high parent expression level and involved in Carbon metabolism, Glycolysis/Gluconeogenesis, and Pyruvate metabolism pathways. Of them, malate synthase1, malate dehydrogenase 2 mitochondrial, and pyruvate kinase were involved in Pyruvate metabolism; alcohol dehydrogenase 1 was involved in Glycolysis/Gluconeogenesis; ADP-ribosylation factor homolog1, citrate synthase1, and glutamate-oxaloacetic transaminase3 were involved in Carbon metabolism. The RT-qPCR results suggested that the expression inheritance of selected genes was strongly consistent with RNA-seq data (Fig.5), indicating that our result is reliable.

## Discussion

Heterosis, or hybrid vigor, was broadly used in breeding programs to improve maize yield, however, its molecular bases are far from clearly described [40, 41]. It is believed that maize heterosis is traits specific since heterosis of one trait cannot serve as an efficient predictor of another trait heterosis [42]. The germination results of our previous study have demonstrated that the seeds showed a superior germination ability in maize hybrid than their parental lines [37]. Similar to our previous results, Guo et al. reported that the significant alterations between hybrids and their parents in protein level might correlate with the superior performance in hybrids [43]. Here, we have demonstrated that the superior performance of hybrids relative to the parental lines might be related to the genes with higher expression level in hybrids.

Gene expression divergence of mRNAs and miRNAs were shown to explain the superior vigor in hybrids compared to their parental lines [44, 45]. It is interesting to find that most differently expressed mRNAs were higher expressed in hybrids, wherever miRNAs were largely repressed [37]. In the present study, the imbibition ability of hybrids was shown to be higher than both parental lines. Similar results were also corroborated by Meyer et al. [46], which stated that hybrids performed better than their parental lines owing to higher gene expression and greater metabolic efficiency during the early stage of germination. In the study researched by Li et al. [34], exogenous abscisic acid (ABA) sensitivity in hybrid B73/Mo17 was lower than in parental inbred lines and the content of abscisic acid in hybrid embryos declined more quickly. The authors stated that the reason for non-ABA sensitivity in hybrid B73/Mo17 is due to the declining ABA gene displaying an above high inbred parent pattern. Consist with this study, the germination results suggest that the genes with high parent dominance pattern or above high parent pattern play important roles in the maize seed germination process.

Although the level of expression divergence between reciprocal hybrids is lower than the comparison of the two parental inbred lines, 501 genes were identified between Z58 × C72 and C72 × Z58. Of these, 173 genes (34.5%) were found to be upregulated and 328 genes (65.5%) were found to be downregulated in Z58 × C72 compared to C72 × Z58. This may also partly explain the reason why the radicle protrusion was initiated earlier in C72 × Z58 than in Z58 × C72 during maize seed germination stage.

Previous studies have suggested that ASE is also considered a mechanism of heterosis. According to the results of a genome-wide analysis of ASE obtained by Shao et al. [24], about 11.5% of the genes showed ASE which may cause dominance or overdominance effects in hybrid. Using RNA-sequencing technology, Hu et al. [29] discovered that 23.8% of expressed genes were identified as being significantly allelic expression and reported a cis-regulatory mechanism of ASE in F_1_ hybrids. In an elite maize hybrid Zhengdan808, 56.4 and 52.4% of analyzed transcripts showed significant allelic expression differences at the spikelet and floret stages, respectively [29]. However, in our study, A total of 116 and 579 ASE genes were obtained in Z58 × C72 or C72 × Z58, respectively. Notably, a subset of the ASE genes, 41 in Z58 × C72 and 108 in C72 × Z58, overlap with the DEGs between the hybrid and parental lines. These ASE genes are mainly driven by cis-regulation. This phenomenon might be related to the mechanism of heterosis during maize seed germination.

Seed germination is a complicated process, which affects crop growth and development and ultimately affects grain yield. Because of the lack of photosynthesis, the initial nutrients are largely dependent on the stored reserves in the seed at the seed germination and post-germinative seedling establishment stage [47]. With absorbing water, the metabolic process of seeds was activated rapidly and provided essential nutrition and energy for seed germination and development [48]. Our transcriptome results suggested that the majority of the DEGs between hybrid and parental lines were involved in metabolic pathways, most of which were mainly enriched in Carbon metabolism, Glycolysis/Gluconeogenesis, and Pyruvate metabolism. Notably, a large number of DEGs involved in metabolic pathways displayed parental dominance up or higher levels expression inheritance. Of them, the gene *Zm00001d003247* encodes malate synthase, involved in pyruvate metabolism and carbon metabolism pathways, which is an important enzyme in the germination process [49]. The gene *Zm00001d032187* encodes malate dehydrogenase, and loss-of-function mutants of its homolog in Arabidopsis showed delayed seed germination rate and frequency [50]. Additionally, several pyruvate kinase genes were classified in parental dominance up or transgressivity up inheritance, such as *Zm00001d026619*, *Zm00001d052494*, and *Zm00001d023379*, whose homologous gene in Arabidopsis was associated with seed germination. And *pkp1*, a loss-of-function mutation of pyruvate kinase in Arabidopsis, has delayed germination compared with wild type [51]. In Arabidopsis, loss of pyruvate kinase activity alters the accumulation of storage oil delaying germination and seedling establishment [52]. Consistent with our present results, Lisec et al. stated that metabolite levels are significant correlate with the biomass heterosis in *Arabidopsis thaliana* [53]. A similar study was also found in soya bean, in which the metabolic genes contribute to heterosis or hybrid vigor [54]. In rubber trees, carbohydrate metabolism is highly associated with growth heterosis, which was further validated by Yang et al. [55]. Using transcriptome and metabolite analysis, Yi et al. revealed that the expression of genes involved in primary and secondary metabolism play important roles in biomass heterosis in rapeseed [56]. In conclusion, we propose that the expression levels of genes related to metabolism in hybrids are higher than those in parental lines and this may promote a faster metabolic rate in hybrids. Further, a faster metabolic rate promotes energy acquisition, cell growth, and reproduction, leading to heterosis.

In this study, we investigated genome-wide gene expression variation of maize embryos in the germination stage of reciprocal hybrids Zhengdan958 and their parental lines. Our research suggests that the majority of differential expression genes were higher in hybrids than in their parental lines. The different expression genes were mainly enriched in metabolic pathways, such as carbon metabolism and glycolysis/gluconeogenesis, which plays important role in germination heterosis. This work provides a comprehensive insight into the molecular mechanisms of heterosis during maize seed germination development.

## Materials and methods

### Maize plants and planting field

Maize hybrid Zhengdan958 is the most widely cultivated hybrid in China, which exhibits high heterosis for grain yield [35]. Together with its parental inbred lines Zheng58 (maternal line, referred to as Z58 hereafter) and Chang7-2 (paternal line, referred to as C72 hereafter) were utilized for this study. The dent inbred line Z58 was selected from a synthetic population of Chinese domestic germplasm with Reid lineage, and the flint inbred line C72 was selected from the Chinese heterotic group Sipingtou [57]. In the summer of 2018, the two parental lines were planted on the farm of Henan agricultural university (Zhengzhou, China). Self-cross and Reciprocal-crosses were made for these two lines.

### Seed germinating condition and sample phenotyping

After harvesting, eighty seeds of the reciprocal hybrids and each parental line were surface-sterilized in 70% (v/v) ethanol for 15 min, followed with rinsed three times with sterile distilled water. For each genotype, three biological replicates were prepared and ten seeds were mixed for each sample. The germinating embryos with 24 hours imbibition at a temperature of 28 °C in an incubator were immediately sampled and stored at − 80 °C for further use.

### RNA extracting and RNA seq (data analysis)

The embryo tissues were peeled from the seeds, and each sample was mixed for immediate RNA extraction. Total RNAs were extracted with TRIzol reagent (Invitrogen) following the manufacturer’s protocol. The total RNAs were processed to reverse transcription reactions to obtain cDNAs followed by the construction of RNA-seq libraries. The same cDNAs were supplied as RT-qPCR templates. To obtain the heterosis-related gene expression, next-generation deep-sequencing was performed. For RNA-seq, 12 libraries were established and accessed by Agilent 2100 Bioanalyzer and ABI StepOnePlus RT-qPCR System for paired-end sequencing with Illumina HiSeq 2500 (Beijing Beirui Biotechnology, Beijing, China).

### RNA-seq data analysis (including QC and DEG)

The raw data obtained by sequencing was filtered using Trimmomatic to remove adapters and low-quality reads [58]. The filtered reads were mapped to the B73 maize reference genome (B73 AGPv4, http://ensembl.gramene.org/ Zea_mays/Info/Index) using Hisat2 [59]. Gene expression was then estimated using StringTie [60], and differential expression analysis of genes was performed using DESeq2 based on gene expression amounts with a fold change > 2 and a false discovery rate (FDR) < 0.05 [61]. The gene ontology (GO) enrichment analysis of these DEGs was performed using agriGO v2.0 (http://systemsbiology.cau.edu.cn/agriGOv2/index.php) with singular enrichment analysis [62]. The Kyoto Encyclopedia of Genes and Genomes (KEGG) enrichment analyses were conducted using the web-based tool KOBAS (http://kobas.cbi.pku.edu.cn/) [63].

### Expression classification

To identify the classification of the different expression genes, DESeq-normalized expression values of each genotype for each gene were obtained. According to previously described by McManus et al. [64], the genes with more than 2-fold expression changes between hybrids and either parent were thought to have a non-conserved inheritance. Based on the Log-transformed values of the ratio of hybrids and parental line, these genes were further classified into eight groups as displaying additive (with Z58 expression lower or higher than C72 expression), Z58 or C72 parental dominance (up and down), and transgressivity (lower or higher than both parent: down and up), respectively. Genes for which expression in the hybrid was higher than Z58 and lower than C72 were classified as additive C72 > Z58 (log_2_^(hyb/Z58)^ > 1, log_2_^(hyb/C72)^ < − 1). Genes for which expression in the hybrid were higher than C72 and lower than Z58 were classified as additive Z58 > C72 (log2(hyb/Z58) < − 1, log2(hyb/C72) > 1). Genes for which expression in the hybrid was higher than Z58 and equal to C72 were classified as C72 dominance up (log_2_^(hyb/Z58)^ > 1, abs(log_2_^(hyb/C72)^) < 1). Genes for which expression in the hybrid was lower than Z58 and equal to C72 were classified as C72 dominance down (log_2_^(hyb/Z58)^ < − 1, abs(log_2_^(hyb/C72)^) < 1). Genes for which expression in the hybrid was higher than C72 and equal to Z58 were classified as Z58 dominance up (log_2_^(hyb/C72)^ > 1, abs(log_2_^(hyb/Z58)^) < 1). Genes for which expression in the hybrid was lower than C72 and equal to Z58 were classified as Z58 dominance down (log_2_^(hyb/C72)^ < − 1, abs(log_2_^(hyb/Z58)^) < 1). Genes for which expression in the hybrid was higher than both C72 and Z58 were classified as transgressivity up (log_2_^(hyb/C72)^ > 1, log_2_^(hyb/Z58)^ > 1). Genes for which expression in the hybrid was lower than both C72 and Z58 were classified as transgressivity down (log2(hyb/C72) < − 1, log2(hyb/Z58) < − 1).

### ASE identification and cis- and trans-regulatory divergence

We used the SNPs between Z58 and C72 based on our previously re-sequencing data as the reference for ASE calling. For the results obtained from the Hisat2 comparison, GATK was used to perform mutation detection. Normalization of trimmed reads number was then performed using function Estimate Size Factors provided by DESeq2 package. For each gene, the ASE was identified by SNPs between two parental genomes. Three thresholds should be met to filter the ASE: (1) Every SNP had at least five reads to support it; (2) There was a significant deviation of the read counts from the two parental alleles; (3) the significant deviation of different SNPs in the same gene was not in different directions. Cis- and trans-regulatory variation were classified according to Bell et al. [65]. In brief, genes were categorized as cis-regulation if the proportion of allele in the F_1_ is biased and the allelic ratio is similar to the parental proportions. Trans-regulatory refers to a balanced allelic expression only in hybrids.

### Gene expression verification

To verify the reliability and reproducibility of the results obtained in the RNA-Seq, some key genes involved in metabolic pathways were selected and further verified by RT-qPCR. The RT-qPCR primers are listed in Table S5. RNA samples were treated with DNase I and further reverse transcribed to cDNA using SYBR Premix Ex Taq II (Tli RNaseH Plus) kit. The RT-qPCR was run on a CFX96 RT-qPCR Detection System. The housekeeping gene *ZmActin1* was set as the endogenous control [66]. The relative expression of each gene was calculated with the 2^-ΔΔCt^ method [67, 68].

## Supplementary Information

**Additional file 1.** Supplementary Information.

**Additional file 2: Table S1.** The inheritance classification and DEGs.

**Additional file 3: Table S2.** The ASE genes and the allelic ratio with statistic test in F1.

**Additional file 4: Table S3.** Distribution of DEGs into different categories of GO in Maize *(Zea mays L.).*

**Additional file 5: Table S4.** The 9 most enriched KEGG pathway terms in Maize *(Zea mays L.)*.

**Additional file 6: Table S5.** The sequence of primers used for RT-qPCR.

**Additional file 7: Fig. S1. A** Germination of inbred lines Z58 and C72 and their F1 hybrids after 48 hours. **B** Germination time course of inbred lines Z58 and C72 and their F1 hybrids.

## Data Availability

The sequence datasets in the fastq format of the current study are available in the NCBI Sequence Read Archive (SRA) database under Bioproject PRJNA788131 (https://www.ncbi.nlm.nih.gov/bioproject/PRJNA788131).

## Abbreviations

DEGs: Differentially expressed genes
ASE: Allele-specific expression
GO: Gene ontology
RT-qPCR: Real-time quantitative PCR
SPE: Specific parental expression
Zheng58: Z58
Chang7-2: C72
