# Supplementary material for: Gene expression variation explains maize seed germination heterosis
Source: BMC Plant Biol. 2022 Jun 20;22:301. doi: 10.1186/s12870-022-03690-x (PMC9208091; doi:10.1186/s12870-022-03690-x)
Supplement: Supplementary file 1 — Additional file 1. Supplementary Information. [file 12870_2022_3690_MOESM1_ESM.doc]

**Supplementary Information**

Gene expression variation explains maize seed germination heterosis

Jiong Wan^1#^, Qiyue Wang^1#^, Jiawen Zhao^1^, Xuehai Zhang^1^, Zhanyong Guo^1^, Desheng Hu^1^, Shujun Meng^1^, Yuan Lin^1^, Xiaoqian Qiu^1^, Liqin Mu^1^, Dong Ding^1*^, Jihua Tang^1*^

^1^National Key Laboratory of Wheat and Maize Crop Science; College of Agronomy; Henan Agricultural University, Zhengzhou 450002, China

^2^The Shennong laboratory, Zhengzhou 450002, China

^#^ Jiong Wan and Qiyue Wang contributed equally to this work.

*Corresponding author

Dong Ding: dingdong0216@hotmail.com

Jihua Tang: tangjihua1@163.com

**Additional file 1:**

**Table S1** The inheritance classification and DEGs.

**Additional file 2:**

**Table S2** The ASE genes and the allelic ratio with statistic test in F1.

**Additional file 3:**

**Table S3** Distribution of DEGs into different categories of GO in **Maize**

***(Zea mays L.)***

**Additional file 4:**

**Table S4** The 9 most enriched KEGG pathway terms in **Maize**

***(Zea mays L.)***

**Additional file 5:**

**Table S5** The sequence of primers used for RT-qPCR.

**Additional file 6:**


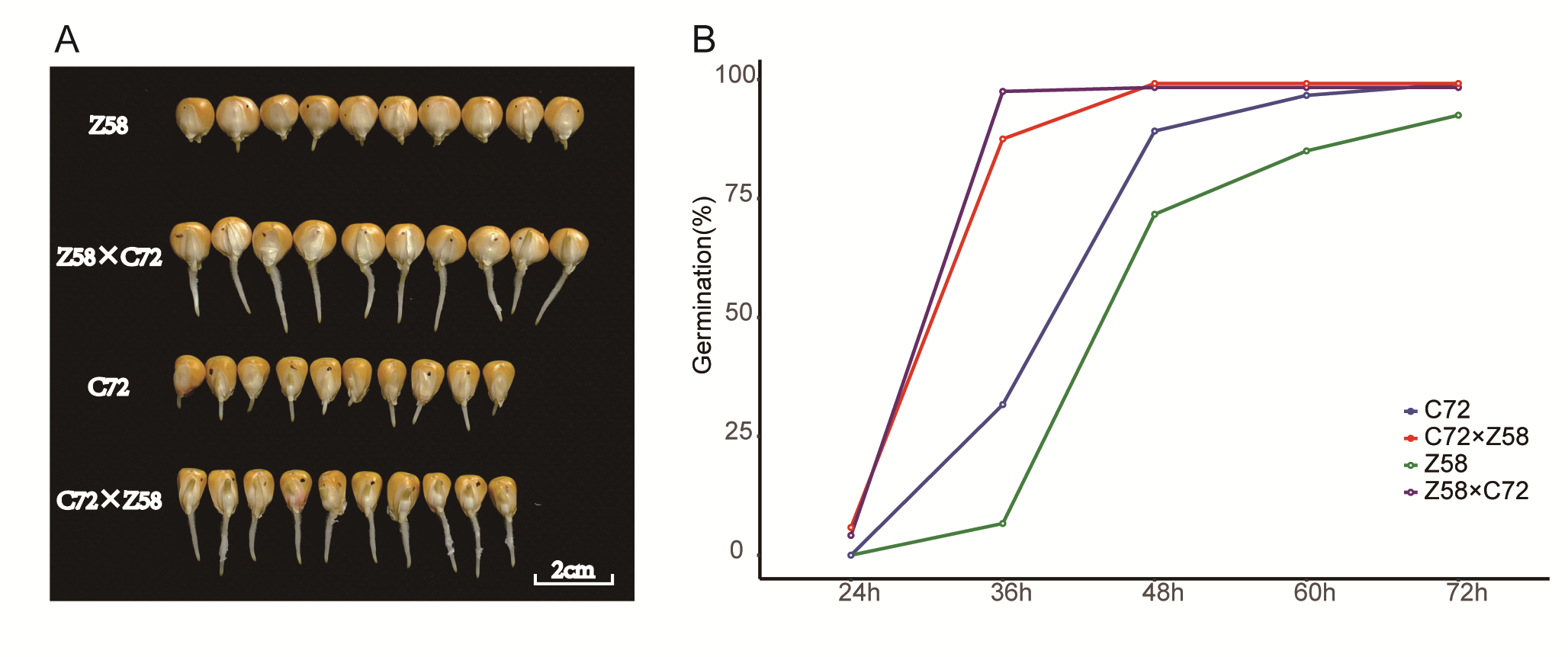


**Figure S1 A** Germination of inbred lines Z58 and C72 and their F1 hybrids after 48 hours. **B** Germination time course of inbred lines Z58 and C72 and their F1 hybrids.
