# Supplementary figures and images for: Gene expression variation explains maize seed germination heterosis
Source: BMC Plant Biol. 2022 Jun 20;22:301. doi: 10.1186/s12870-022-03690-x (PMC9208091; doi:10.1186/s12870-022-03690-x)

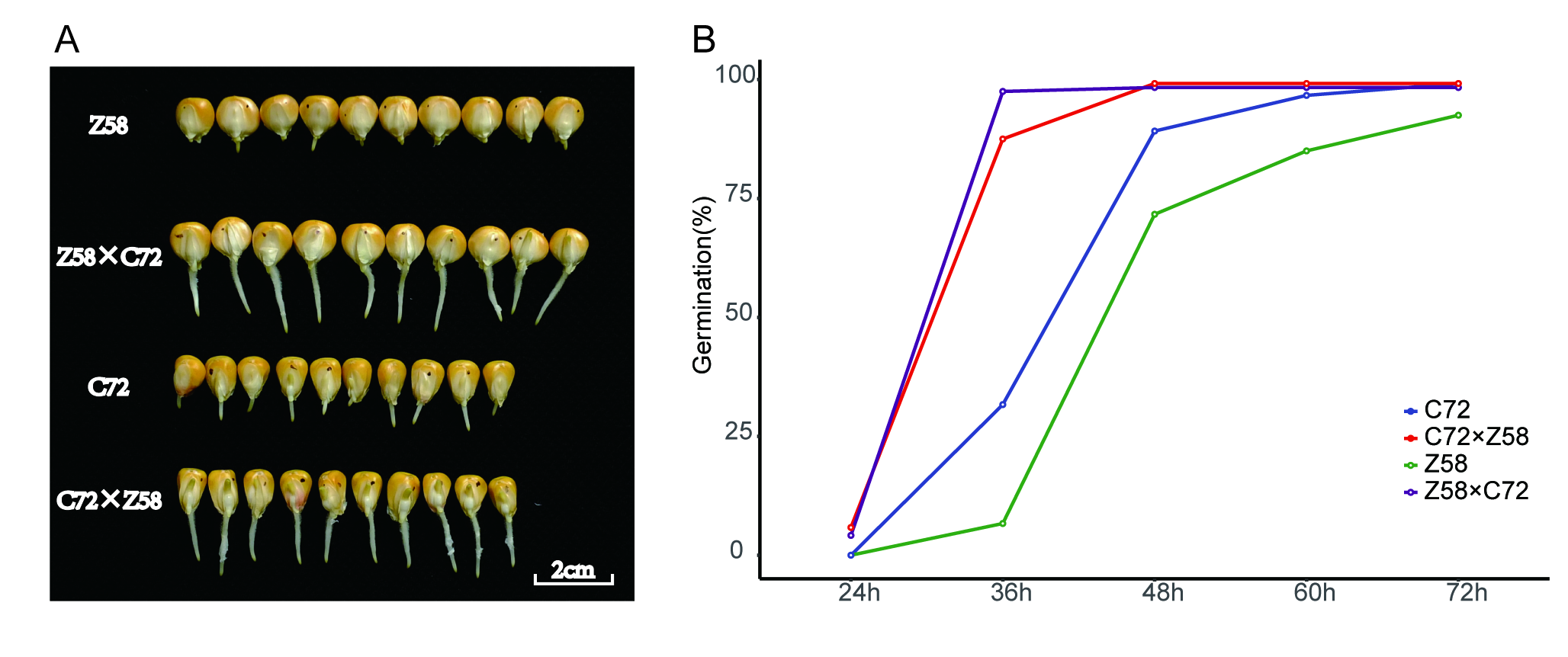

Supplement: Supplementary file 7 — Additional file 7: Fig. S1. A Germination of inbred lines Z58 and C72 and their F1 hybrids after 48 hours. B Germination time course of inbred lines Z58 and C72 and their F1 hybrids. [file 12870_2022_3690_MOESM7_ESM.tif]
